# Methods for Assessing and Optimizing Solar Orientation by Non-Planar Sensor Arrays

**DOI:** 10.3390/s19112561

**Published:** 2019-06-05

**Authors:** Jiang Wang, Xingang Fan, Yongchao Zhang, Jianyu Yang, Yuming Du, Jianxin He

**Affiliations:** 1 School of Communication and Information Engineering, University of Electronic Science and Technology of China, Chengdu 610054, Sichuan, China; rivers2000@126.com (J.W.); jyyang@uestc.edu.cn (J.Y.); 2 School of Electronic Engineering, Chengdu University of Information Technology, Chengdu 610225, Sichuan, China; dym@cuit.edu.cn (Y.D.); hjx@cuit.edu.cn (J.H.); 3 Department of Geography and Geology, Western Kentucky University, Bowling Green, KY 42101, USA; xingang.fan@wku.edu

**Keywords:** non-planar sensor array, orientation determination, performance assessment and optimization

## Abstract

Non-planar sensor arrays are used to determine solar orientation based on the orientation matrix formed by orientation vectors of the sensor planes. Solar panels or existing photodiodes can be directly used without increasing the size or mass of the spacecraft. However, a limiting factor for the improvement of the accuracy of orientation lies with the lack of an assessment-based approach. A formulation was developed for the supremum (i.e., the least upper bound) of orientation error of an arbitrary orientation matrix in terms of its influencing factors. The new formulation offers a way to evaluate the supremum of orientation error considering interference with finite energy and interference with infinite energy but finite average energy. For a given non-planar sensor array, a sub-matrix of the full orientation matrix would reach the optimal accuracy of orientation if its supremum of orientation error is the least. Principles for designing an optimal sensor array relate to the configuration of the orientation matrix, which can be pre-determined for a given number of sensors. Simulations and field experiment tested and validated the methods, showing that our sensor array optimization method outperforms the existing methods, while providing a way of assessment and optimization.

## 1. Introduction

Determination of solar orientation is a critical technique that has a wide range of applications, including spacecraft attitude estimation [1,2] assisted positioning for planetary rovers [3] ground-based navigation systems [4] and efficiency improvement of solar power plants [5]. In the aerospace fields that require high accuracy solar orientation, sun sensors based on optical imaging are used, such as complementary metal-oxide semiconductors [6,7] charge-coupled-devices [8] and micro-electro-mechanical systems [9,10]. These sensors usually consist of a set of optical and mechanical elements, and measure the image position of the Sun on the planar array through a small masking hole/slit. Given the finite geometric heights of the masking hole/slit above the planar array, the detectable field of view of these sensors is less than 180${}^{\circ }$. Therefore, at least three such sun sensors are required to determine the Sun’s position for full view-field applications. However, this requirement leads to an increased load for small aerospace equipment such as nano-satellites, which is problematic because of their limited size, weight, and power supply.

Another primary method in solar orientation determination is based on non-planar sensor arrays that are formed by photodiodes equipped on different surfaces of spacecrafts [1,2,11] or sometimes by the direct re-use of solar panels of the spacecrafts [12]. However, due to its susceptibility to interference and its lack of accuracy assessment when interference occurs, this method is primarily limited to ground tracking of the Sun [5] and to spacecrafts that do not require high accuracy in solar orientation. Thus, the ideal solution must meet both the navigational needs and sizing limitations of small-scale aerospace equipment while also maintaining a highly accurate orientation of the Sun, improving the accuracy of orientation of the non-planar sensor array method could be one way to achieve this balance.

One major source of error in solar orientation determination using non-planar sensor arrays comes from the interference of radiation reaching the sensors. The interference may originate from misalignments and undesired scale factor of photodiodes, and interfering light sources from surrounding environment such as scattered and reflected sunlight [13,14,15,16]. In an effort to improve the accuracy of orientation determination, methods for calibrating misalignments and undesired scale factor of photodiodes have been developed to suppress the output error of photodiodes [13,14]. These calibrations enable higher accuracy in solar orientation determination with a given hardware. However, the orientation determination error caused by the same interference varies with and depends on the configuration of sensor arrays [15,16,17]. Therefore, it could be difficult to achieve high solar orientation accuracy by using these calibrations if the sensor array is not configured well.

Optimal design and configuration for non-planar sensor arrays are additional ways to improve the accuracy of solar orientation. Many non-planar sensor arrays have been designed for determining the solar orientation, examples include sensor arrays in the shapes of a hemisphere [4], cube [11], two triangular pyramid [18], and truncated pyramid [17,19]. For better configuring sensor arrays, two optimal design methods have been developed based on the minimum estimated variance [15] and bias [16] of the unit vector pointing towards the Sun, respectively. However, there is a lack of common evaluation method for various sensor array configuration parameters. For example, the solar orientation can be determined by three illuminated photodiodes with non-coplanar normal vectors [4]. If more than three photodiodes are illuminated and all have non-coplanar normal vectors, then multiple solar orientation directions can be obtained. The difficulty is then to determine which one would result in the highest accuracy. Reference [19] provides two formulations for assessing the errors in determination of the solar azimuth and elevation angles, which can be used to optimize the sensor array configuration parameters. However, these formulations are limited to truncated pyramid sensor arrays. Thus, there is a need to find an assessment-based approach for the optimal design of the array configuration parameters.

This study addresses the above problems by first establishing a mathematical formulation for the orientation error in terms of its influencing factors; then, proposing an assessment method by using the defined error formulation; and finally, putting forward an optimization method, which outperforms the existing optimization methods of sensor array design and configuration. The rest of this article is organized as follows: Section 2 introduces the method for orientation determination of the Sun based on non-planar sensor arrays; Section 3 establishes the relationship between the orientation error and its influencing factors; Section 4 and Section 5 present the orientation performance assessment and optimization, respectively; Section 6 provides verification of the assessment and optimization method by means of simulation and field experiment; Section 7 concludes this study with summary and discussions.

## 2. Method for Orientation Determination Based on Non-Planar Sensor Arrays

Because the Sun is far enough from the observation site, the Sun’s rays reaching the observation site can be assumed to be parallel to each other. Therefore, we define the Sun vector to be pointing towards the Sun from the observation point with a magnitude equaling its irradiance. Please note that the Sun vector in this work is defined as the opposite direction of sun’s rays, while the Sun vector defined in [4] is its unit vector.

**Figure 1:**
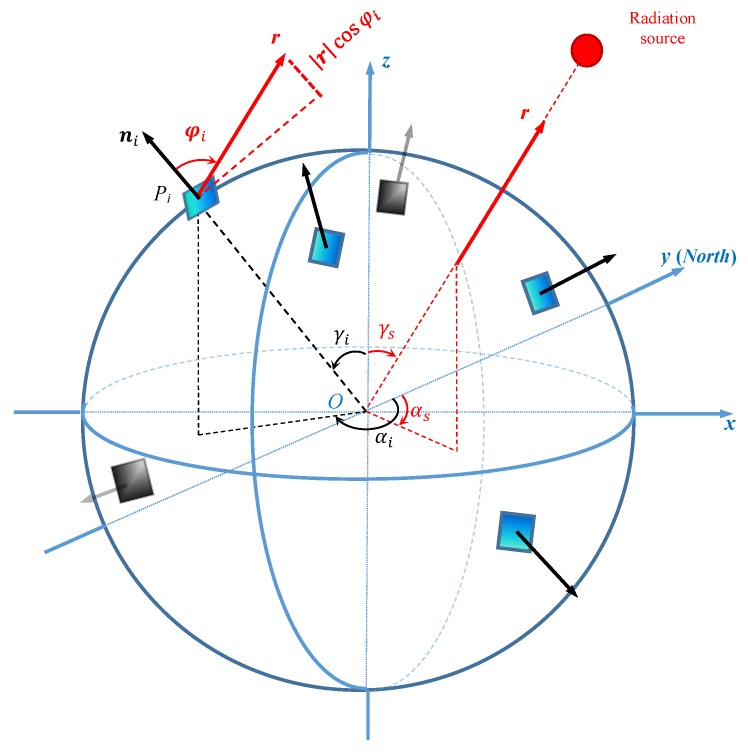
Geometric relationship of the Sun vector and the sensor array in an sphere coordinate system, where the sensor array aligns with the x-y-z coordinate of the spacecraft, the Sun vector r has an azimuth angle $\alpha_{s}$ and zenith angle $\gamma_{s}$, the sensor plane $P_{i}$ has an azimuth $\alpha_{i}$ and zenith $\gamma_{i}$, the unit normal direction of the sensor plane $n_{i}$ points towards local vertical direction with an angle $\varphi_{i}$ between r and $n_{i}$.

The geometric relationship between the Sun vector and the sensor array system is shown in Figure 1. The Cartesian coordinate system x-y-z aligns with the sensor array illustrated on an sphere, with the observation point at the origin O. In the system, the Sun vector r has an azimuth angle $\alpha_{s}$ and zenith angle $\gamma_{s}$. The illuminated sensor plane $P_{i}$ (where $i\in \left\{1,2,\ldots ,M\right\},M\geq 3$) is mounted at azimuth $\alpha_{i}$ and zenith $\gamma_{i}$; its unit normal vector ni aligns with local vertical, forming an angle $\varphi_{i}$ with r. Azimuth angle is the angle from the true north (if applied on the Earth), here set as the positive y direction, and rotating to east to a projected vector on the x-O-y plane. Zenith angle is the angle between a vector and the positive *z* direction. According to the cosine law for radiation [20]—the irradiance that passes vertically through the sensor plane is $\left|r\right|\cos\varphi_{i}$ ($\left|r\right|$ is the length of the vector r), and consequently, the output currents of photodiode sensors, for instance, vary with $\cos\varphi_{i}$. Please note that the irradiance passing through the sensor plane is equal to the inner product of the Sun vector and the unit normal vector of the sensor plane $r\cdot n_{i}=\left|r\right|\cos\varphi_{i}$.

For the irradiance passing through a sensor $P_{i}$, the output measurement value $e_{i}$ usually relates linearly to the input by a factor $\eta_{i}$ and can be assumed to be

(1) $$e_{i}=\frac{1}{\eta_{i}}\left|r\right|\cos\varphi_{i}$$

Thus the irradiance passing through the sensor is $\left|r\right|\cos\varphi_{i}=\eta_{i}e_{i}$. Then we further obtain the following matrix equation for the sensor array:

(2) $$\left(\begin{array}{c} n_{1}^{T}\\ n_{2}^{T}\\ \vdots \\ n_{M}^{T} \end{array}\right)r=\left(\begin{array}{c} \eta_{1}e_{1}\\ \eta_{2}e_{2}\\ \vdots \\ \eta_{M}e_{M} \end{array}\right)$$

where $n_{i}={\left(\begin{array}{ccc} \sin\alpha_{i}\sin\gamma_{i} & \cos\alpha_{i}\sin\gamma_{i} & \cos\gamma_{i} \end{array}\right)}^{T}$ and $r=\left|r\right|{\left(\begin{array}{ccc} \sin\alpha_{s}\sin\gamma_{s} & \cos\alpha_{s}\sin\gamma_{s} & \cos\gamma_{s} \end{array}\right)}^{T}$ according to the geometric relationships shown in Figure 1.

In Equation (2), ${\left(\begin{array}{cccc} n_{1} & n_{2} & \cdots & n_{M} \end{array}\right)}^{T}$ is the orientation matrix formed by the unit normal vectors of all *M* illuminated sensor planes, if denoted by A, then we have

(3) $$A=\left(\begin{array}{ccc} \sin\alpha_{1}\sin\gamma_{1} & \cos\alpha_{1}\sin\gamma_{1} & \cos\gamma_{1}\\ \sin\alpha_{2}\sin\gamma_{2} & \cos\alpha_{2}\sin\gamma_{2} & \cos\gamma_{2}\\ \vdots & \vdots & \vdots \\ \sin\alpha_{M}\sin\gamma_{M} & \cos\alpha_{M}\sin\gamma_{M} & \cos\gamma_{M} \end{array}\right)$$

So, Equation (2) becomes

(4) $$\mathrm{Ar}=\left(\begin{array}{c} \eta_{1}e_{1}\\ \eta_{2}e_{2}\\ \vdots \\ \eta_{M}e_{M} \end{array}\right)$$

Assuming that the unit normal vectors of the *M* sensor planes are non-coplanar, the rank of A is equal to the number of its columns, i.e., rank(A)=3. Then, there is a unique solution for the matrix equation:

(5) $$r={\left(A^{T}A\right)}^{-1}A^{T}\left(\begin{array}{c} \eta_{1}e_{1}\\ \eta_{2}e_{2}\\ \vdots \\ \eta_{M}e_{M} \end{array}\right)$$

For a sensor array that use similar sensors, the measurement coefficients $\eta_{i}$ may be reasonably assumed to be equal to a constant $\eta \left(\eta >0\right)$. If we denote the measurement vector as $e={\left(\begin{array}{cccc} e_{1} & e_{2} & \cdots & e_{M} \end{array}\right)}^{T}$, the Sun vector solution can be simplified as

(6) $$r=\eta {\left(A^{T}A\right)}^{-1}A^{T}e$$

The orientation of a sun vector is determined by three component vectors in a three dimensional space. Thus, by using Equation (6), the direction of the Sun can be determined if there are three or more illuminated non-coplanar sensors. It is worth noting that when M>3, the direction of the Sun can be determined either by the full orientation matrix A or by any of its sub-matrices as long as there are at least three non-coplanar sensors included in the sub-matrix.

The discussion from hereon will consider, as in any practical applications, an orientation matrix $H_{m\times 3}$, which is either the full orientation matrix A or any of its sub-matrix that is formed by *m* illuminated sensors, where m≤M. Accordingly, the measurement vector e will have only *m* elements. Similar to Equation (6), now

(7) $$r=\eta {\left(H^{T}H\right)}^{-1}H^{T}e$$

## 3. Mathematical Formulation of Orientation Error

As mentioned earlier, the irradiance measured in practical applications always contains influence of interferences. Assume that an interference vector ε is added to the measurement vector e. According to Equation (7), the estimated sun vector, denoted by $r^{\prime }$, has the form:

(8) $$r^{\prime }=\eta {\left(H^{T}H\right)}^{-1}H\left(e+\varepsilon \right)$$

The error of the estimated sun vector, i.e., the difference between $r^{\prime }$ and r, is

(9) $$\Delta r=\eta {\left(H^{T}H\right)}^{-1}H^{T}\varepsilon$$

Since the column vectors of H belong to m-dimensional linear real space $R^{m}$ and are linearly independent of each other, the column space constructed by the column vectors of H is a closed subspace of $R^{m}$. According to the orthogonal decomposition theorem of vectors, there is a unique pair of orthogonal vectors v and u in $R^{m}$ that satisfy ε=v+u, where v is the component vector of ε projected in the column space of H and u is the component vector of ε projected in the orthogonal complementary space. Since the column vectors of H are orthogonal to vector u and thus $H^{T}u=0$, by substituting ε=v+u into Equation (9) we have:

(10) $$\Delta r=\eta {\left(H^{T}H\right)}^{-1}H^{T}\left(v+u\right)=\eta {\left(H^{T}H\right)}^{-1}H^{T}v$$

Equations (9) and (10) suggest that the estimation error of sun vector is dependent only on the component of the interference vector in the column space of the orientation matrix H, and not on the orthogonal component.

**Figure 2:**
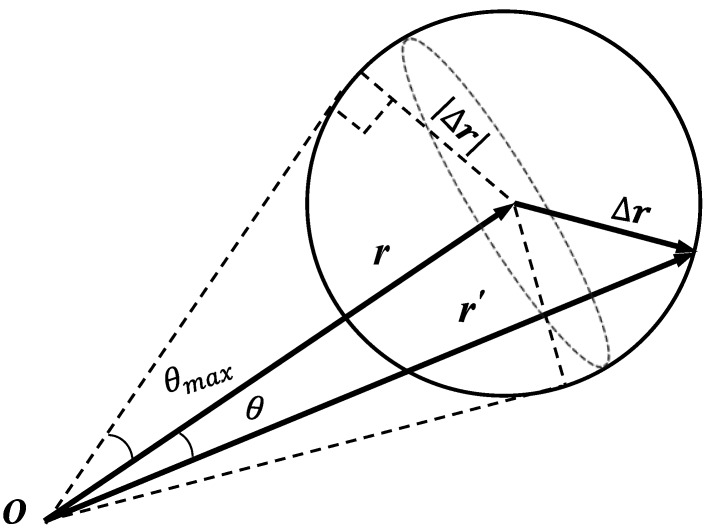
Geometric relations between the orientation error θ and the vectors r, $r^{\prime }$, and Δr.

The orientation error can be measured by the angle θ between the Sun vector r and its estimate $r^{\prime }$ [17], as shown in Figure 2. The angle θ varies with the vector estimation error Δr. When $\left|\Delta r\right|<\left|r\right|$ and Δr is perpendicular to $r^{\prime }$, the orientation error θ is the largest (denoted as $\theta_{\max}$) for even the same $\left|\Delta r\right|$; when Δr is of the same or opposite direction of r, θ is zero. According to the geometric relationship in Figure 2, when $\left|\Delta r\right|<\left|r\right|$ and Δr is satisfied, θ can be written as:

(11) θ≤θmax=arcsinΔrΔrrr

Since ${∥Tx∥}_{2}\leq {∥T∥}_{2}{∥x∥}_{2}$, when *T* is a bounded linear operator and ${∥x∥}_{2}=\left|x\right|$ for the real vector x with the Euclidean norm ${∥•∥}_{2}$, from Equation (10), we know that $\left|\Delta r\right|<\eta {∥{\left(H^{T}H\right)}^{-1}H^{T}∥}_{2}\left|v\right|$, and consequently, we obtain:

(12) Δrr≤HTH−1HT2vrrηη

Then, Equation (11) becomes:

(13) θ≤θmax=arcsinHTH−1HT2vrrηη

The assumption $\left|\Delta r\right|<\left|r\right|$ results in θmax<ππ22 according to Equation (11); and the input of arcsine function should satisfy HTH−1HT2vrrηη<1. Usually, $\left|v\right|$ is far less than rrηη, and when ${∥{\left(H^{T}H\right)}^{-1}H^{T}∥}_{2}$ is small, HTH−1HT2vrrηη<1 is easily satisfied.

According to the property of singular values, the non-zero singular value of H is the positive square root of the corresponding non-zero eigenvalue of matrix $H^{T}H$ or $HH^{T}$. Similarly, the non-zero singular value of matrix ${\left(H^{T}H\right)}^{-1}H^{T}$ is the positive square root of the non-zero eigenvalue of matrix ${\left(H^{T}H\right)}^{-1}H^{T}{\left({\left(H^{T}H\right)}^{-1}H^{T}\right)}^{T}$. ${\left(H^{T}H\right)}^{-1}H^{T}{\left({\left(H^{T}H\right)}^{-1}H^{T}\right)}^{T}$ is equal to ${\left(H^{T}H\right)}^{-1}$ and the eigenvalue of ${\left(H^{T}H\right)}^{-1}$ and that of matrix $H^{T}H$ are the inverse of each other, which suggests that the non-zero singular value of matrix ${\left(H^{T}H\right)}^{-1}H^{T}$ and that of H are also the inverse of each other.

Assume that the minimum non-zero singular value of H is $\sigma_{\min}$. Since the spectral norm of the matrix is equal to its maximum non-zero singular value, HTH−1HT2=11σminσmin. Usually, v is unknown, assume v2v2ε2ε2=μ, from Equation (13) we obtain the following orientation error formulation:

(14) θ≤arcsinμσminε2rrηη

where ε is the interference vector; rrηη relates to the measurement value of the irradiance; $\sigma_{\min}$ is the minimum singular value of the orientation matrix H; and μ is the ratio between the projected energy ${\left|v\right|}^{2}$ of the interference in the orientation matrix column space; and ${\left|\varepsilon \right|}^{2}$ is the interference energy, which is determined by the interference vector and the orientation matrix H. Equation (14) indicates that the orientation error relates to three influencing factors: the interference vector, the orientation matrix, and the irradiance measurement. More importantly, Equation (14) gives the definition of the supremum (i.e., the least upper bound) of the orientation error, which can be expressed as:

(15) θsup=arcsinμσminε2rrηη

In applications, the ratio μ is usually unknown. According to the Pythagorean theorem, the interference vector ε and its orthogonal component vector v satisfy ${\left|v\right|}^{2}\leq {\left|\varepsilon \right|}^{2}$, Thus μ has an upper bound value of one, i.e., μ≤1. When the interference vector is fully projected onto the column space of the orientation matrix, μ is equal to 1. For the convenience of the following discussions, we denote the $\theta_{\sup}$ under full impact of the interferences (i.e., when μ=1) as $\theta_{\sup\_fi}$, which is termed as full impact supremum hereafter:

(16) θsup_fi=arcsin1σminε2rrηη

## 4. Assessment of Orientation Determination

In the orientation determination of the Sun, the smaller the full impact supremum of the orientation error as expressed in Equation (16), the smaller the maximum value of the orientation error caused by any interference, and thus, the higher the accuracy of orientation will be in the application. Therefore, the full impact supremum of the orientation error of the Sun defined in Equation (16) can be used to assess the orientation performance. During the observation, the intensity of the Sun $\left|r\right|$ can be assumed constant, and the measurement ratio η is also constant. In the following, we discuss the assessment of the orientation determination error in two scenarios of interference energy ${\left|\varepsilon \right|}^{2}$: interference with finite energy and interference with infinite energy but finite average energy, along with $\sigma_{\min}$, which is determined by the orientation matrix.

In practical applications, interference is generated by undesirable sensor scale factor, assembly error of sensor planes on the non-planar array, multipath propagation, imperfect surrounding measurement circuitry, and other radiation sources [16]. Considering that the radiation energy of the Sun is finite, and the law of energy conservation, the interference energy can be reasonably assumed finite on each sensor plane. Even in the cases when one or several sensors fail, the total interference energy can still be assumed finite. This assumption means that ${\left|\varepsilon \right|}^{2}$ in Equation (16) is finite. The full impact supremum of the orientation error relates to 11σminσmin, with the given sensor planes and the interference. For the convenience of discussion, we define 11σminσmin as an interference coefficient and denote it with κ. This interference coefficient κ is solely dependent on the orientation matrix by its minimum singular value $\sigma_{\min}$. It functions as a magnifying factor that relates the full impact supremum of orientation error of a given orientation matrix to the given interference that has finite energy. Thus, the orientation performance of the Sun can be evaluated using the interference coefficient κ=11σminσmin. A larger minimum singular value $\sigma_{\min}$, i.e., smaller interference coefficient κ, suggests a smaller full impact supremum of orientation error and better orientation performance. It is summarized as:

(17) $$\sin\theta_{\sup}\propto \kappa$$

The second scenario with regard to the interference energy is the case of infinite total energy but finite average energy. In cases where *m* or *M* increase to a very large number, total interference energy from all sensors may continue to increase, but we can still assume the average interference energy is finite. For example, the interference caused by atmospheric scattering in clear-sky causes an interference energy on every sensor. Then, the total interference energy from all sensors may become infinite with m→∞, but we can assume the average interference energy is finite, i.e., ε2ε2mm is finite. Similar to the above discussion, we can define an average interference coefficient, κa=mmσmin=κmσmin=κm. Thus, the orientation performance of the Sun can be evaluated using the average interference coefficient in the scenario of interference with infinite energy but finite average energy. Similarly, a smaller average interference coefficient suggests better orientation performance, i.e.,

(18) $$\sin\theta_{\sup}\propto \kappa_{a}$$

## 5. Optimization of Orientation Determination

In both scenarios of interference, as discussed in the previous section, the orientation error is ultimately determined by the orientation matrix. However, different orientation matrices (e.g., sub-matrices by combining different sensors) might result in varied interference coefficients. Therefore, the optimization of the orientation determination can be realized by selecting the right orientation matrix that minimizes the interference coefficients κ or $\kappa_{a}$, leading to the minimization of the orientation error.

The orientation matrix H has the size of m×3, of which all the row vectors are unit vectors. So, the trace of $H^{T}H$ is equal to *m*. In addition, the property of singular values suggests that the trace of matrix $H^{T}H$ is equal to the quadratic sum of the singular values of matrix H. Thus, the quadratic sum of the singular values of the orientation matrix H is equal to *m*. Considering that the orientation matrix H has three non-zero singular values and their quadratic sum is *m*, its minimum non-zero singular value σmin≤mm33. Please note that the maximum possible value of $\sigma_{\min}$ (i.e., σmin≤mm33 is realized only when all three non-zero singular values are equal. For the orientation matrix H, the range of the interference coefficient κ is

(19) κ=11σmin≥33mmσmin≥33mm

Equation (19) indicates that the minimum κ is simply inversely proportional to $\sqrt{m}$. Therefore, in cases of finite total interference energy, the optimal sensor array will have as many as possible illuminated sensors, and the three non-zero singular values of their orientation matrix should be equal.

Similarly, in cases of finite average interference energy, the range of the average interference coefficient $\kappa_{a}$ is

(20) κa=mmσmin≥3σmin≥3

From Equation (20), the minimum $\kappa_{a}$ is the constant $\sqrt{3}$ and is achieved when the three non-zero singular values are equal. It is not related to the number of sensors involved in the orientation matrix.

In summary, in a given field of view, to minimize κ and $\kappa_{a}$ and to achieve highest accuracy of orientation, the design of the sensor array should follow the following principles: (1) all sensor planes of the array are illuminated by the Sun; and (2) the nonzero singular values of the orientation matrix composed of all sensor planes are equal.

## 6. Applications and Analysis

### 6.1. Simulations

According to the method discussed above and other exiting methods for array design, the sensor array for orientation determination can be designed and optimized by three principles: (1) based on the minimum full impact supremum of orientation error (this study); (2) based on the minimum estimated variance of the Sun vector [15]; and (3) based on the minimum estimated bias of the Sun vector [16]. In this section, we design five sensor arrays to validate the orientation error formulation and the method for orientation error assessment put forward in this study. Their orientation performance is also compared through simulations.

**Figure 3:**
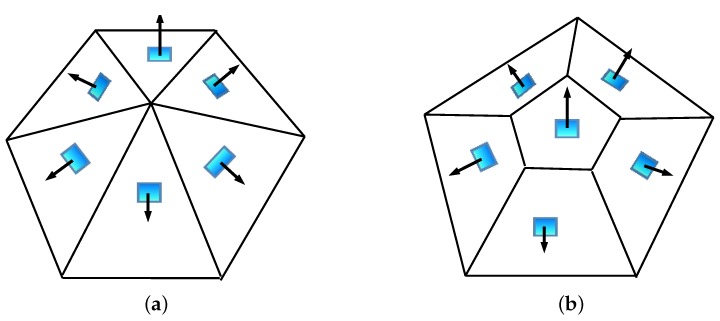
(**a**) The schematic image of arrays 1 and 3, and (**b**) the schematic image of Arrays 2, 4, and 5.

**Figure 4:**
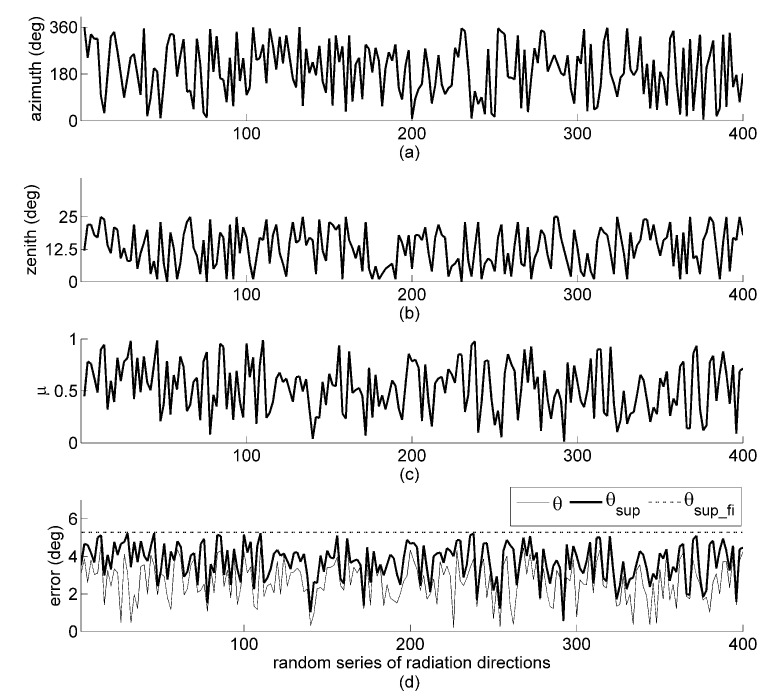
(**a**) Azimuth angle, (**b**) zenith angle, (**c**) ratio μ of the projected interference energy, and (**d**) the resulting orientation error θ, supremum $\theta_{\sup}$, and full impact supremum $\theta_{\sup\_fi}$.

The configurations of the five chosen sensor arrays, each formed by six sensor planes, including the azimuth and zenith angles of each unit normal vector of the sensor planes and the non-zero singular values of the orientation matrix, are given in Table 1. Array 1 and Array 3 consist of all sides of hexagonal pyramid and the schematic image is shown in Figure 3a. The other three arrays consist of all lateral and top faces of the truncated pentagonal pyramid and the schematic image is shown in Figure 3b. Array 1 is designed without considering any optimization methods. Array 2 and Array 3 are two different arrays designed by following the minimum full impact supremum of orientation error with all six sensor planes illuminated. Array 4 is adapted from [16] and designed based on the minimum estimated variance of the Sun vector with all six sensor planes illuminated. Array 5 is also adapted from [16], which is designed based on the minimum estimated bias of the Sun vector with all six sensor planes illuminated. In order for all the sensor planes in the above five arrays to be illuminated, the solar zenith angle is limited to the 0 to 25${}^{\circ }$ range, but the solar azimuth angle can vary randomly from 0 to 360${}^{\circ }$, as shown in Figure 4a,b. Due to the intensity of the Sun $\left|r\right|$ and the fact that the measurement ratio η of similar sensors can be assumed constant, we set an arbitrary value for $\left|r\right|=100$ and η=1 during the simulations.

As discussed earlier, out of the six sensor planes in each of the sensor array in Table 1, many different orientation matrices can be formed by 3–6 sensor planes. By following the principles provided in Section 5 for designing sensor arrays, we first identify the orientation matrix that has the minimum κ and $\kappa_{a}$, denoted as $\kappa_{\min}$ and $\kappa_{a\_\min}$, respectively, for each sensor array. From the results shown in Table 2, the full orientation matrix of all sensor arrays has the minimum κ and $\kappa_{a}$, except for Array 1, for which the minimum $\kappa_{a}$ is found in a submatrix that uses only five sensor planes. With the best orientation matrix found, the second step for validating the orientation error formulations is to calculate the measurement vector e by Equation (1), interference vector ε (discussed below), and estimated sun vector $r^{\prime }$ by Equation (8), for a given sun vector r.

For the interference vector ε, we consider two situations in terms of how it is projected. First, the interference energy is randomly projected onto the column space of the orientation matrix while keeping the total energy ${\left|\varepsilon \right|}^{2}$ constant. The interference vector ε is generated by two steps: (1) generate a normally distributed pseudorandom numbers with the length of *m*; and (2) make the square sum of pseudorandom numbers equal to ${\left|\varepsilon \right|}^{2}$. Second, the interference vector is fully projected onto the column space of the orientation matrix, i.e., μ=1, while the total energy ${\left|\varepsilon \right|}^{2}$ is still kept constant. In this situation, the interference vector ε is generated by three steps: (1) generate a normally distributed pseudorandom numbers with the length of 3; (2) generate a vector by combining the column vectors of the orientation matrix using the three pseudorandom numbers as the coefficients; (3) adjust the length of the combined vector to be equal to $\left|\varepsilon \right|$.

In generating the random interference projections on each sensor in this study, the normally distributed pseudorandom numbers vary with orientation matrix and direction of the Sun. We used randomly generated solar directions for simulation, as shown in Figure 4a,b, the random series of azimuth and zenith angles. Figure 4c shows the actual μ values when the interference vector is randomly projected onto the column space of Array 1’s orientation matrix and the interference energy ${\left|\varepsilon \right|}^{2}$ is set to 100, which is arbitrarily set to an equivalent level as the Sun. The result of orientation determination by the full orientation matrix of sensor Array 1 is shown in Figure 4d. The orientation error θ is always smaller than the supremum $\theta_{\sup}$ from Equation (15), and the supremum $\theta_{\sup}$ never exceeds the full impact supremum $\theta_{\sup\_fi}$. These results support the validness of the orientation error formulations.

For validating the influence of κ and $\kappa_{a}$ on the orientation performance, we consider two scenarios of interference energy as discussed in Section 4. First, for finite interference energy, we assume that all orientation matrices in Table 2 contain a faulty sensor and the interference energy ${\left|\varepsilon \right|}^{2}$ caused by the faulty sensor is arbitrarily set to 100, which is equivalent to the energy level of the Sun. Second, for interference with infinite energy but finite average energy, we assume that the average interference energy ε2ε2mm on the sensor planes of any orientation matrices in Table 2 is arbitrarily set to 16.67 (=100/6). From the results shown in Table 3, the orientation matrix with $\kappa_{\min}$ showed the smallest $\theta_{\sup\_fi}$ in the context of interference with the same total energy. The orientation matrix with $\kappa_{a\_\min}$ showed the smallest $\theta_{\sup\_fi}$ in the context of interference with the same average energy. In addition, the results also indicate that the optimal orientation matrix is not unique, as Arrays 2 and 3 gave the same results. These results validate the assessment methods of orientation performance.

**Figure 5:**
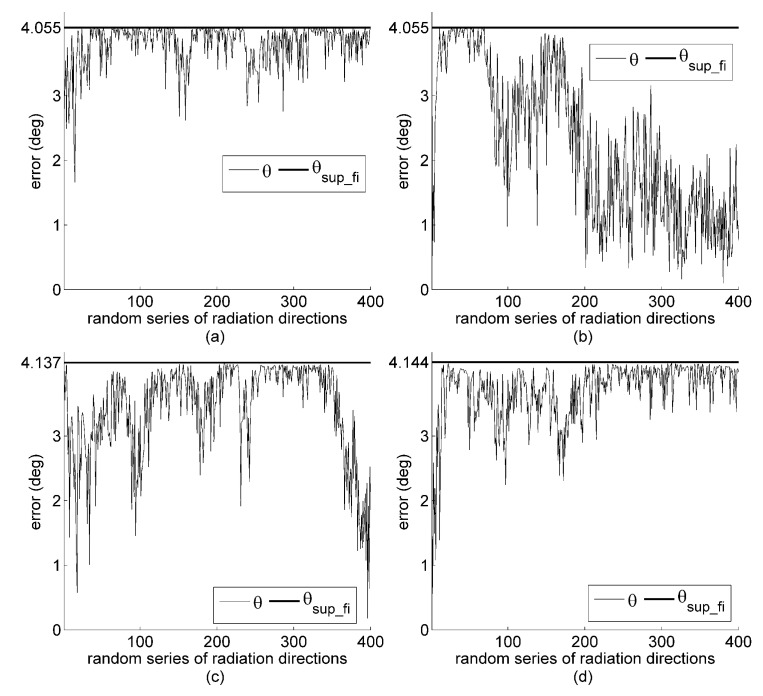
Simulated orientation error of sensor Arrays 2, 3, 4, and 5 in (**a**–**d**) under full interference impact when μ=1 but with the same series of solar positions as in Figure 4.

In an overall examination of the herein proposed design, assessment, and optimization methods, Arrays 2–5 are simulated in the context of interference energy being fully projected onto the column space of the orientation matrix (i.e., μ=1) and the total energy set to 100 (or average energy set to 16.67). In the simulation, the direction of Sun and projection of the interference energy vary randomly. The simulation results for determining the direction of the Sun are shown in Figure 5 for the four sensor arrays simulated. The results indicate that, with a given number of sensors for an array, the orientation matrix with the smallest $\theta_{\sup\_fi}$ resulted in more superior orientation performance. According to the error formulations in Equations (15) and (16), the $\theta_{\sup\_fi}$ of the Array 4 and Array 5 are larger than that of Arrays 2 and 3. This result validates our claim that the optimization method proposed in this study outperforms the ones based on estimated variance or bias. In addition, it is worth noting that the method of this study can now assess the performance of Arrays 4 and 5. The orientation performance of the design based on smallest estimated variance is better than that based on the smallest estimated bias.

### 6.2. Field Experiment of Solar Orientation Determination

**Figure 6:**
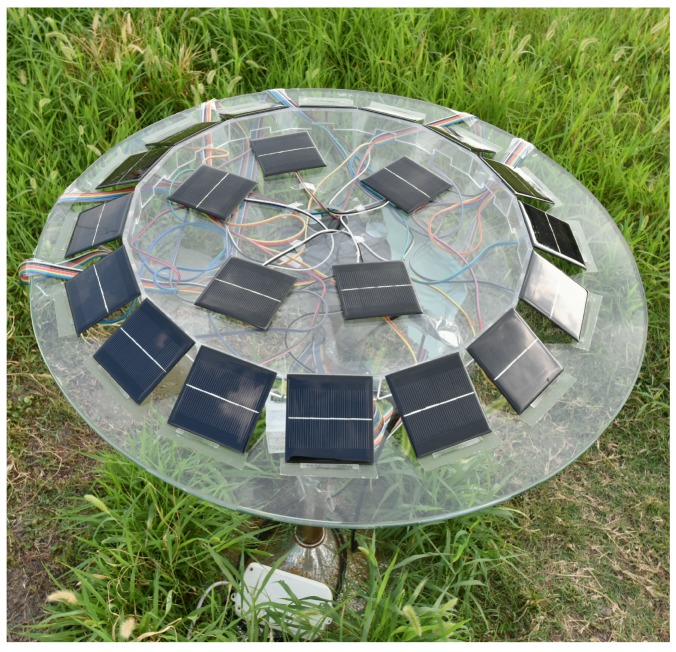
The sensor array for determining the solar orientation.

Our solar sensor array is designed such that 16 solar panels are mounted on the lateral surfaces of a regular 16-pyramid, which is placed on a rotating horizontal glass tabletop (Figure 6). For increasing the detectable field of view, the angle between the lateral and bottom faces of the pyramid is designed to be 26.4${}^{\circ }$. The solar panels used are monocrystalline silicon batteries with 5 V open-circuit voltage and 160 mA short-circuit current. With the same irradiance, the output error of the solar panels is <±5%. The assembly error of various solar panels is <±1${}^{\circ }$. These errors, which vary with the true output of the solar panel, can be added as a disturbance in the output measurements of each solar panel to interfere with the solar orientation determination. Since the short-circuit current outputs of the solar panels vary approximately linearly with illumination intensity, the solar irradiance on various surfaces of the pyramid is measured by the short-circuit current output of the solar panels. In addition, five solar panels mounted on the top of the pyramid in a horizontal plane parallel to the ground surface are used to measure the total solar irradiance on the ground by the average of their short-circuit current outputs.

For calculating the position of the Sun, a Cartesian coordinate system is set with the center of the pyramid base as the origin, the ground surface as the *x*-y coordinate plane with *y*-axis pointing towards true north and *x*-axis towards east. In setting up the sensor array, one side of the regular 16-pyramid is arbitrarily selected to align with one side of the compass with error <0.5${}^{\circ }$ first, and then rotate the horizontal tabletop until the compass points towards the geographical south. In the system, the true azimuth angle $\alpha_{s}$ and zenith angle $\gamma_{s}$ of the Sun are calculated using the following astronomical formulas

(21) $$\begin{array}{cc} \cos\left(\gamma_{s}\right) & =\sin\left(\psi \right)\sin\left(\delta \right)+\cos\left(\psi \right)\cos\left(\delta \right)\cos\left(t\right) \end{array}$$

(22) $$\begin{array}{cc} \sin\left(\alpha_{s}\right) & =-\frac{\cos\left(\delta \right)\sin\left(t\right)}{\sin\left(\gamma_{s}\right)} \end{array}$$

where ψ is local latitude and the solar hour angle *t* and declination angle δ can be calculated according to the astronomical algorithm [21]. The astronomical algorithm is valid from 1901 to 2099. The calculating errors of the algorithm to *t* is <±30 s and δ is <±0.03${}^{\circ }$, which ensures high accuracy of $\alpha_{s}$ and $\gamma_{s}$ calculated by Equations (21) and (22). The unit vector of the true sun vector can be expressed as $\left(\begin{array}{ccc} \sin\alpha_{s}\sin\gamma_{s} & \cos\alpha_{s}\sin\gamma_{s} & \cos\gamma_{s} \end{array}\right)$, and solar orientation errors can be calculated by the inverse cosine of the inner product of this vector and the unit vector of the Sun vector measured by our sensor.

**Figure 7:**
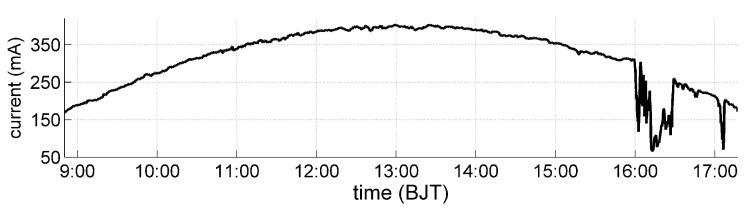
The total solar irradiance measured by the average output current (mA) of the five solar panels mounted on top of the pyramid.

The field experiment was conducted in the suburb of Chengdu, China (longitude 103${}^{\circ }$59’ east, latitude 30${}^{\circ }$35’ north). The measurement platform was installed at the meteorological observation site with a broad field of view. The measurement was carried out once every second on 15 August 2015, with sunny weather in the morning, thin clouds around the Sun after 1525 BJT (Beijing Time), and thin clouds covering the Sun at 1545–1630 BJT and around 1707 BJT. All solar panels were illuminated by sunlight within the solar azimuth angle ranged from 86.8 to 271.7${}^{\circ }$ during the period of observation, while the zenith angle ranged from 60.1 to 13.3${}^{\circ }$ and then returned to 57.4${}^{\circ }$. The total solar irradiance changed in a cosine waveform with respect to solar altitude angle $\left(90-\gamma_{s}\right)$${}^{\circ }$ and dropped sharply in the cloudy periods (1600–1630 and around 1707 BJT) as shown in Figure 7, which was consistent with the actual weather conditions.

With the given 16 solar panels, we calculated κ and $\kappa_{a}$ values of all possible orientation matrices formed by any combinations of three or more non-coplanar solar panels. Among all possible orientation matrices, 15 have small κ and $\kappa_{a}$ values and their configurations are given in Table 4. Please note that the zenith angles of all solar panels are 26.4${}^{\circ }$, while their azimuth angles increase from zero at 22.5${}^{\circ }$ increments. All 15 orientation matrices listed in Table 4 have $\kappa_{a}=3.18$, while κ varies from 1.59, 1.12, 0.92, to 0.8 when different number of solar panels are used (m = 4, 8, 12, 16, respectively).

**Figure 8:**
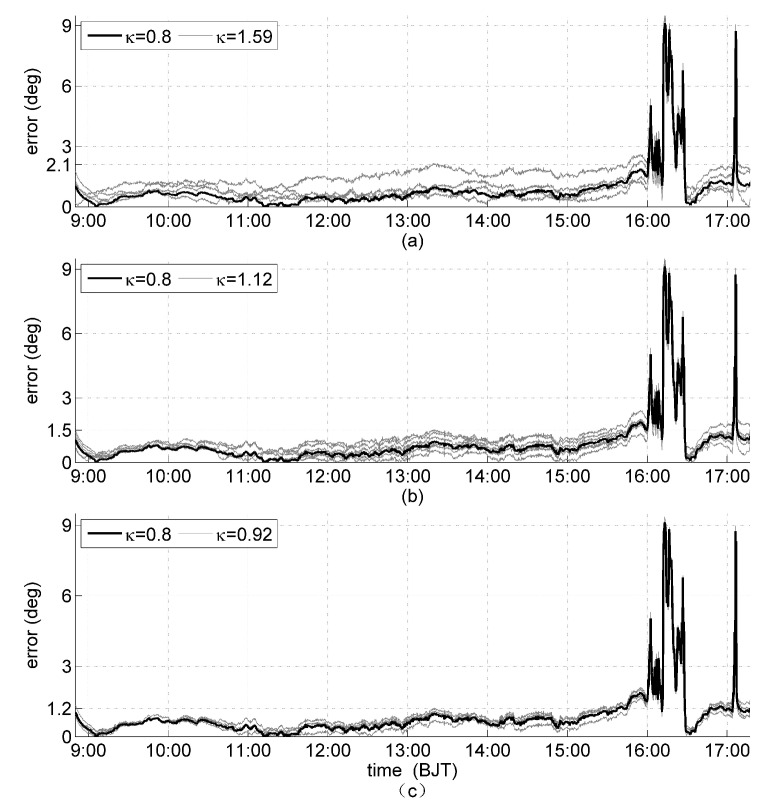
Orientation error for orientation matrices with the same $\kappa_{a}$ but different κ values: κ = 1.59, 1.12, and 0.92 shown as thin gray lines in (**a**–**c**); and κ = 0.8 as thick black line in all cases.

**Figure 9:**
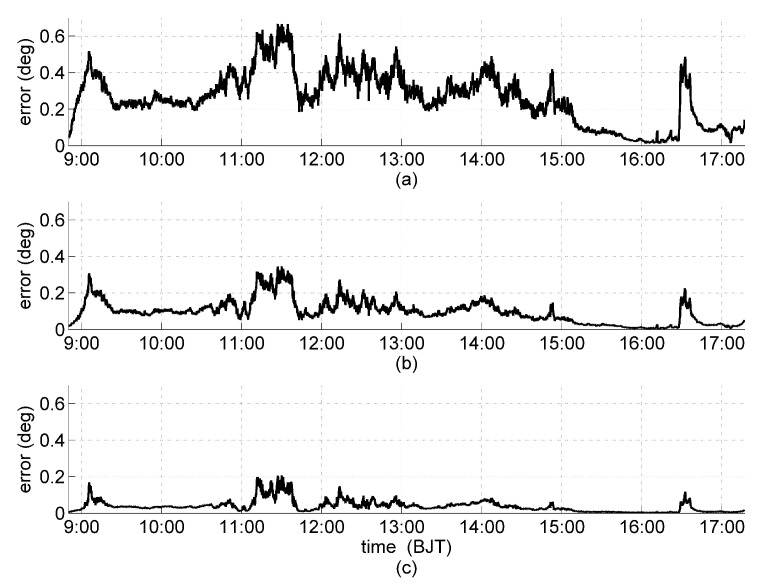
Difference of the average orientation error of orientation matrices that have the same κ and the matrix 15 whose κ = 0.8 is the smallest: (**a**) κ = 1.59 and κ = 0.8; (**b**) κ = 1.12 and κ = 0.8; and (**c**) κ = 0.92 and κ = 0.8.

The solar orientation was determined every 10 s by using all 15 orientation matrices in Table 4 and the orientation error analysis are shown in Figure 8. Orientation errors of the orientation matrices that have the same $\kappa_{a}$ are grouped together. The bold line is the orientation error of matrix 15 with the minimum κ and $\kappa_{a}$ ($\kappa_{\min}=0.8$, $\kappa_{a\_\min}=3.18$), and each gray line is a different configuration among the ones with the same *k* value. The maximum orientation error during the clear-sky period are 2.1, 1.5, and 1.2${}^{\circ }$ for κ = 1.59, 1.12, and 0.92, respectively (Figure 8a–c). It is obvious that the smaller the κ, the smaller the orientation error. To illustrate this more clearly, the difference between the average orientation error from all the orientation matrices that have the same κ and the orientation error of matrix 15 (κ = 0.8) is shown in Figure 9. First, the all-positive differences reveal that at each time point of observation, matrix 15 has the smallest error compared to other matrices of larger κ values. Second, with decreasing κ, the orientation error also decreases (Figure 9a–c). These results show that the orientation matrix 15 with $\kappa_{\min}$ and $\kappa_{a\_\min}$ has the most superior orientation performance in practical applications. This conclusion is consistent with the theoretical analysis and simulation results.

Furthermore, during the clear-sky periods, sine of the maximum orientation errors is approximately proportional to κ. For the results shown in Figure 8, sin2.1∘sin2.1∘1.59≈1.59≈sin1.5∘sin1.5∘1.12≈1.12≈sin1.2∘sin1.2∘0.92≈0.92≈0.02. This again confirms the validity of [20]. During the cloudy periods, the interference energy on each solar panel is primarily generated by atmospheric scattering and are approximately equal. Thus, the orientation errors of all 15 orientation matrices with the same $\kappa_{a}$ are also approximately equal, which validates [21].

## 7. Conclusions

In this study, we developed a theoretical formulation for the supremum of the orientation error of an arbitrary orientation matrix with more than three non-coplanar sensors. With the formulation, the supremum of orientation error in situations of interference with finite energy or interference with infinite energy but finite average energy can be evaluated. The orientation performance relates to the number of sensor planes and the minimum non-zero singular value of the orientation matrix. Based on the minimum non-zero singular values, the interference coefficient and average interference coefficient were defined for a given orientation matrix.These coefficients can be used in assessment of a sensor array design. For a given number of non-planar sensors, principles for designing the optimal sensor array are found to be the configuration of an orientation matrix to reach the minimum supremum of the orientation error among all possible orientation matrices. Particularly in a given field of view, when the full orientation matrix formed by all sensors has the same nonzero singular values, the sensor array can achieve the optimal orientation determination.

The optimal design of the sensor array based on our theory can improve the overall performance of solar orientation determination than the design based on estimated variance or bias. The optimal sensor array of various constructions can be easily designed by using the orientation matrices that are non-unique and can be calculated accurately with the same smallest supremum of orientation error. Simulations and our field experiment confirmed that the solar orientation performance is only determined by the average interference coefficient in case of interference with infinite energy but finite average energy. Among the orientation matrices with the same average interference coefficient, the smaller the interference coefficient, the better the orientation performance. For improving the accuracy of orientation in the scenario of the unknown interference energy distribution, the orientation matrix with the minimum interference coefficient and minimum average interference coefficient should be selected.

## Figures and Tables

**Table 1:** Configuration of the selected sensor arrays.

Array	Zenith Angle (deg)						Azimuth Angle (deg)						Non-zero Singular Values of Orientation Matrix Formed by All Six Sensor Planes		
	1	2	3	4	5	6	1	2	3	4	5	6	1	2	3
1	40	45	45	45	45	45	90	18	306	234	162	150	1.8	1.25	1.09
2	63.435	63.435	63.435	63.435	63.435	0	90	18	306	234	162	0	1.41	1.41	1.41
3	54.736	54.736	54.736	54.736	54.736	54.736	90	30	330	270	210	150	1.41	1.41	1.41
4	60	60	63	65	64	0	339	266	195	52	124	93	1.45	1.40	1.39
5	65	60	65	65	0	60	143	27	100	172	164	314	1.44	1.42	1.38

**Table 2:** Minimum values of interference coefficient κ and average interference coefficient $\kappa_{a}$ for the orientation matrices formed by *m* sensor planes.

Array	Orientation Matrix with $\kappa_{\min}$		Orientation Matrix with $\kappa_{a\;\min}$	
	$\kappa_{\min}$	m	$\kappa_{a\;\min}$	m
1	0.9199	6	2.0733	5
2	0.7071	6	1.7321	6
3	0.7071	6	1.7321	6
4	0.7214	6	1.7669	6
5	0.7227	6	1.7702	6

**Table 3:** The full impact supremum of orientation error $\theta_{\sup\_fi}$.

Array	Interference with the Same Total Energy on the Sensorplanes Forming the Orientation Matrix (i.e., ${\left|\varepsilon \right|}^{2}=100$)		Interference with the Same Average Energy on the Sensor Planes Forming the Orientation Matrix (i.e., ${\left|\varepsilon \right|}^{2}/m=16.67$, where *m* is Number of Sensor Planes Forming the Orientation Matrix)	
	$\theta_{\sup\_f_{i}}$ of the Matrix with $\kappa_{\min}$	$\theta_{\sup\_f_{i}}$ of the Matrix with $\kappa_{a\;\min}$	$\theta_{\sup\_f_{i}}$ of the Matrix with $\kappa_{\min}$	$\theta_{\sup\_f_{i}}$ of the Matrix with $\kappa_{a\;\min}$
1	5.278	5.320	5.278	4.856
2	4.055	4.055	4.055	4.055
3	4.055	4.055	4.055	4.055
4	4.137	4.137	4.137	4.137
5	4.144	4.144	4.144	4.144

**Table 4:** The configuration of 15 orientation matrices and their κ and $\kappa_{a}$, with lit solar panels marked by black dot.

Matrix	Azimuth Angle (deg) of the Solar Panels																	
	0	22.5	45	77.5	90	112.5	135	157.5	180	202.5	225	247.5	270	292.5	315	337.5	κ	$\kappa_{a}$
1	•				•				•				•					
2		•				•				•				•			1.59	
3			•				•				•				•			
4				•				•				•				•		
5	•	•			•	•			•	•			•	•				
6	•		•		•		•		•		•		•		•			3.18
7	•			•	•			•	•			•	•			•	1.12	
8		•	•			•	•			•	•			•	•			
9		•		•		•		•		•		•		•		•		
10			•	•			•	•			•	•			•	•		
11	•	•	•		•	•	•		•	•	•		•	•	•			
12	•	•		•	•	•		•	•	•		•	•	•		•	0.92	
13	•		•	•	•		•	•	•		•	•	•		•	•		
14		•	•	•		•	•	•		•	•	•		•	•	•		
15	•	•	•	•	•	•	•	•	•	•	•	•	•	•	•	•	0.8	
